# Influence of linear intravenous energy density on the success of intravenous laser ablation for treatment of chronic venous insufficiency

**DOI:** 10.1590/1677-5449.190009

**Published:** 2019-08-29

**Authors:** Luiza de Freitas Heineberg, Adriana Buechner de Freitas Brandão, Filipe Carlos Caron, Jaqueline Beirith, Walter Junior Boim de Araujo

**Affiliations:** 1 Faculdades Pequeno Príncipe, Curso Superior de Medicina, Curitiba, PR, Brazil.; 2 Instituto da Circulação - Excelência em Angiologia, Cirurgia Vascular e Endovascular, Departamento de Cirurgia, Curitiba, PR, Brazil.

**Keywords:** venous insufficiency, laser, saphenous vein, varicose veins

## Abstract

**Background:**

It is relevant to elucidate the influence that mean linear endovenous energy density (LEED) has on the success of endovenous laser ablation treatment for chronic venous insufficiency, in order to reduce the method’s adverse effects.

**Objectives:**

To evaluate the influence of mean LEED on the prevalence of saphenous closure 30 days after the laser ablation procedure.

**Methods:**

153 lower limbs from 118 patients seen at a tertiary hospital and treated for chronic venous insufficiency with endovenous 1470 nm laser ablation under local anesthesia were evaluated. The mean LEED used to treat patients was calculated to determine whether greater than average LEED was required for treatment success.

**Results:**

A significant difference (p = 0.021) in saphenofemoral junction closure was associated with mean LEED used above the knee. Conversely, there was no significant difference in the thigh segment.

**Conclusions:**

Linear intravenous energy density greater than the mean of 70.57 J/cm was associated with a higher rate of closure at the saphenofemoral junction. However, density did not have an influence on the result for the thigh segment, showing that an energy density exceeding 70.57 J/cm tends not to be required for treatment of this segment.

## INTRODUCTION

Chronic venous insufficiency (CVI) is a common disease in the vascular surgeon’s office, affecting 25 to 33% of women and 10 to 20% of men in the adult Western population.^1^ It is directly related to work absenteeism and to public healthcare expenditure.^2^ Considering this socioeconomic importance, studying new techniques that can enable effective treatment with better results is generating increasing interest, particularly with relation to rates of relapse, to complications, and to early return to daily activities.^3^

Treatments for CVI include: clinical management and lifestyle changes; foam sclerotherapy and varicose vein surgery using the mini-incisions technique; saphenectomy by fleboextraction; and endoluminal methods such as radio frequency and laser thermoablation (endovenous laser ablation, EVLA).^4^^-^ 7

The minimally invasive techniques are alternatives to conventional surgical treatment with great saphenous vein (GSV) stripping and they have been increasingly adopted within medical practice.^5^^,^ 6 Of these, EVLA stands out for its favorable results in terms of saphenous closure rates, patient satisfaction, and reduced post-procedure morbidity.^3^^,^ 8 ^,^ 9

In view of the growing acceptance of EVLA as an alternative method to stripping for treatment of CVI,^10^ this study was conducted with the intention of elucidating what influence linear endovenous energy density (LEED) has on the outcome of the procedure at 30 days.

## METHODS

This was a retrospective study, that reviewed medical charts for 193 patients with CVI seen at a tertiary hospital, treated with EVLA, and reassessed 30 days later. The project was approved by the institutional Ethics Committee and registered under protocol number 80030817.0.00005580 on the Plataforma Brasil.

The inclusion criteria were patients with CVI treated with EVLA from the saphenofemoral junction (SFJ) to the knee at the Hospital Angelina Caron from 2011 to February of 2018. Exclusion criteria were: patients who did not return for the 30-day follow-up; patients with peripheral arterial disease, severe hypercoagulation syndromes, or a history of deep venous thrombosis or deep venous insufficiency; and patients with incomplete medical records.

A total of 153 lower limbs were analyzed from 118 patients who had been treated with EVLA for CVI. The procedure was initiated with the patient in decubitus dorsal. Anesthetic was applied to the puncture site and ultrasound-guided puncture of the GSV was conducted using an Abocath^®^16, preferably at a point distal of the last tributary vein with reflux, followed by insertion of a 6F valved introducer. Next, the optical fiber (radial or linear) was inserted and connected to an endovenous laser energy source with a standard wavelength of 1470 nm (registered with the Agência Nacional da Vigilância Sanitária, under number 80058580018).

**Figure 1:**
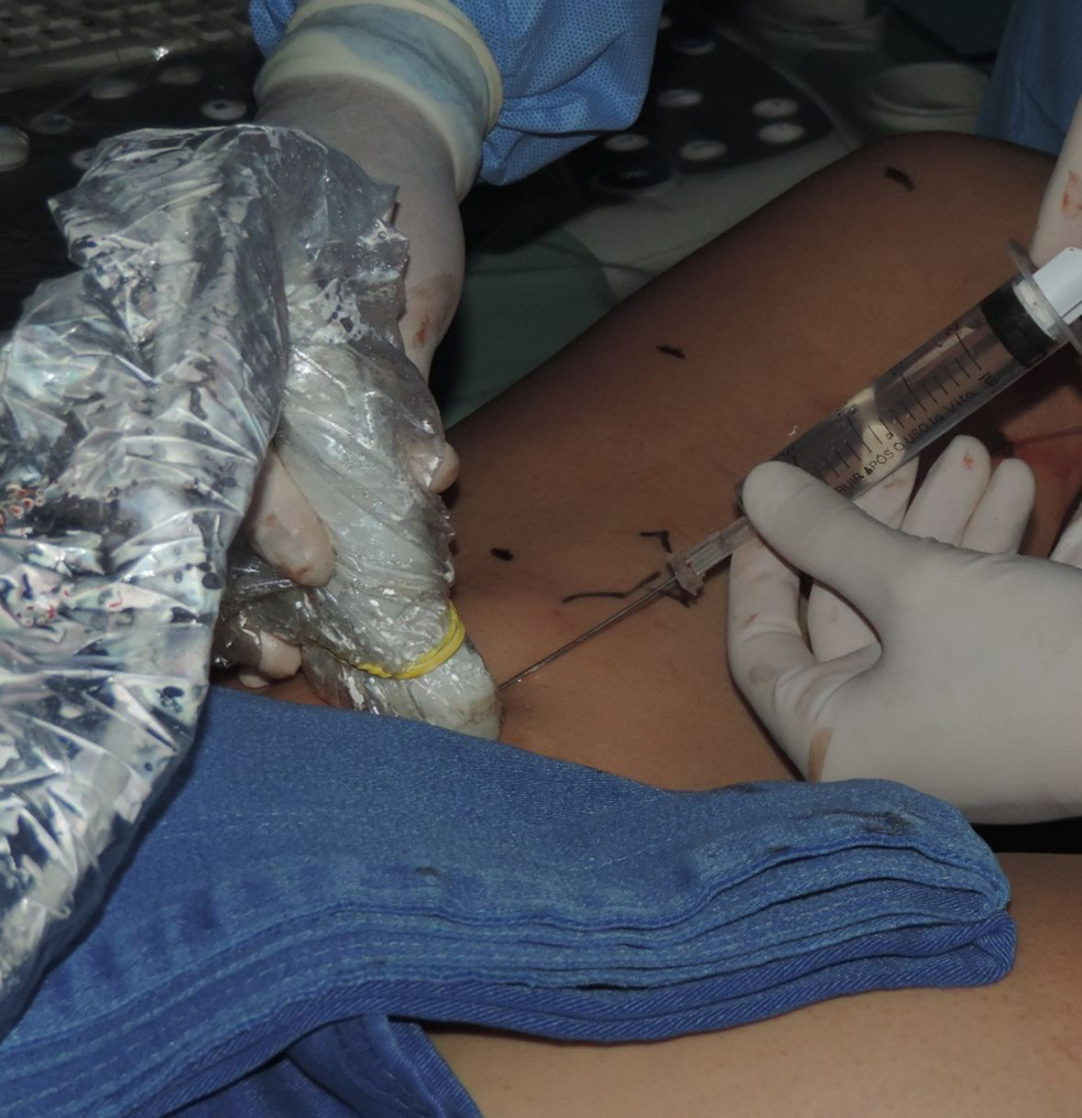
Initiating tumescent anesthesia induction with ultrasound control.

**Figure 2:**
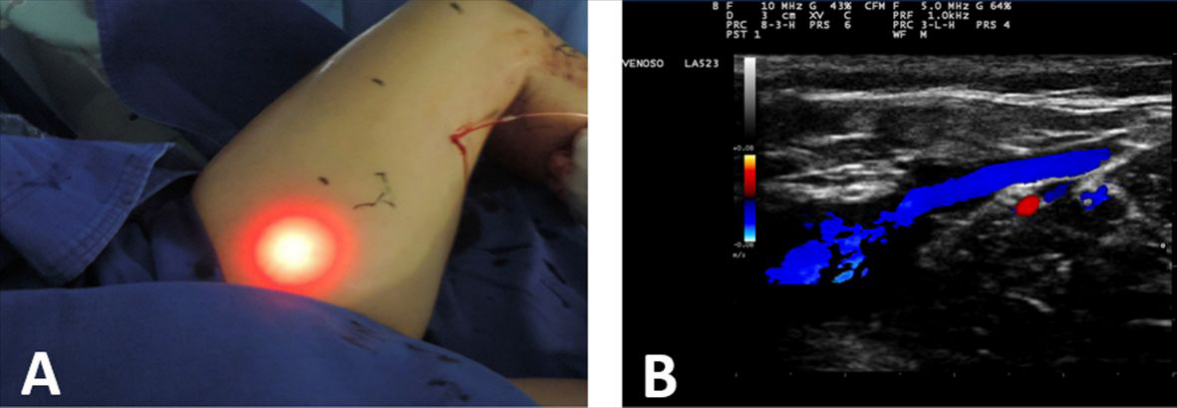
Confirmation of fiber position by transillumination (A) and ultrasound control (B).

Once the endoluminal position within the GSV at 2.5 cm distal of the SFJ had been confirmed, tumescent local anesthesia was administered along the course of the GSV being treated. A syringe or mechanical infusion pump was used to administer a solution comprising 250 mL 0.9% saline to 20 mL of 2% lidocaine and adrenaline 1:100,000 with 4 mL of 8.4% sodium bicarbonate (Figure 1). Once anesthesia was complete, the position of the fiber at 2.5 cm from the junction was confirmed once more and the laser activated (Figure 2). The fiber was tractioned manually and continuously, in the caudal direction up to the distal limit of the saphenous vein being treated.^11^

After the procedure, dressing was applied, exerting extrinsic compression along the path of the GSV, using cotton wool pads and 7/8 high compression elastic stockings (30-40 mmHg). Patients were discharged from hospital on the same day as the operation, after approximately 2 hours of postoperative observation, with a 5-day prescription for analgesics and nonsteroidal anti-inflammatories. The elastic stockings were left in place for 48 hours and removed by the patients themselves. From the third day onwards, the patient wore the stockings during the day and removed them for bathing and sleeping. Although there were no specific restrictions on walking, patients were advised to spend around 40 minutes of each hour at rest with legs raised.

**Figure 3:**
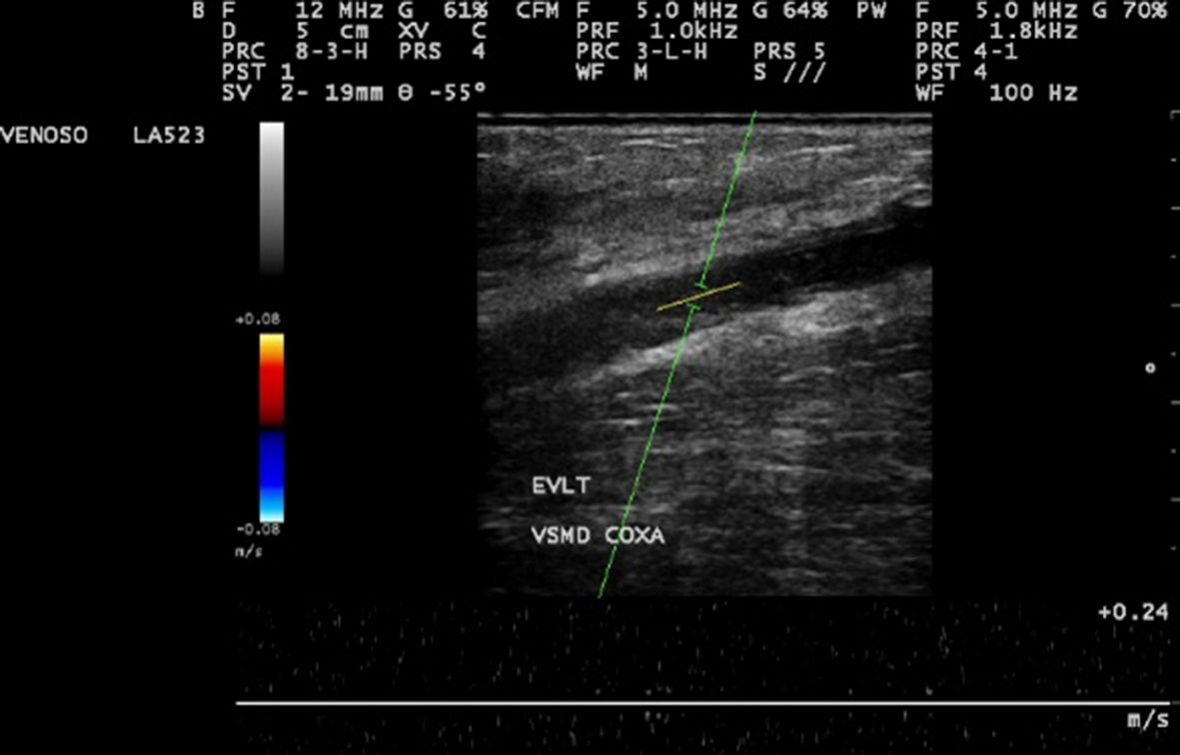
Control ultrasound examination at 7 postoperative days, demonstrating absence of flow in the saphenous vein.

Patients were reassessed at 7 and 30 days after surgery, with physical examination and Doppler vascular echography (Figure 3). The most important factors analyzed were: presence or absence of paresthesia, phlebitis, and deep venous thrombosis in the treated lower limb and the rates of GSV obliteration and absence of reflux.

Ultrasound assessment of absence of reflux at the SFJ followed the recommendations contained in the clinical practice guidelines for patients with varicose veins and venous diseases published by the Society for Vascular Surgery and the American Venous Forum. A positive result was defined as absence of patent GSV stump and no reflux. For analysis, patients were classified as absence of patent stump, partially patent stump, or presence of reflux (Table 1).

**Table 1:** Classification of results of Doppler ultrasonography at the saphenofemoral junction after thermal ablation.

Patency	J0	Absence of patent stump
	J1, J2, J3, J4, etc.	Junction with 1, 2, 3, 4 cm, etc. patent stump
Reflux	R+	Reflux
	R-	No reflux

### Statistical analysis

Data were collected from medical records and stored in a Microsoft Excel spreadsheet. Data analysis was conducted with the help of SPSS, version 22.0.

Results were expressed using descriptive statistics. Inferential analysis used Pearson’s chi-square test, Fisher’s exact test, Student’s *t* test for parametric variables and the Mann-Whitney test for nonparametric variables. Results with p values below 0.05 were considered significant.

## RESULTS

A total of 193 patient records were analyzed and 118 records and 153 lower limbs were selected for analysis. The majority of the study population were female (55.9%) and overweight (mean body mass index [BMI] was 29.67] and the average age was 55.35 years. A two-ring radial fiber was used in 66.8% and a linear fiber was used in 33.2% of the patients, both with a 600 µ diameter, and power was 6 watts. Descriptive statistics for the data are shown below in tables, by age and BMI (Table 2), by sex (Table 3), and by complications (Table 4).

**Table 2:** Descriptive statistics for age and BMI data.

**Statistical analysis**	**Age (years)**	**BMI (kg/m^2^)**
n	118	118
Mean	52.35	29.67
Standard deviation	11.74	5.57

BMI, body mass index.

**Table 3:** Descriptive statistics for sex.

**Sex**	**Frequency**	**Percentage**
Female	66	55.9
Male	52	44.1
Total	118	100.0

**Table 4:** Descriptive statistics for data on postprocedural complications in 118 patients.

**Complication**	**Frequency**	**Percentage**
DVT	1	0.8
Paresthesia	31	26.3
Phlebitis	16	13.5

DVT, deep venous thrombosis.

There were significant differences between the outcome groups (absence of patent stump, partially patent stump, and presence of reflux) with relation to mean LEED administered, according to analysis of variance (ANOVA), with p = 0.021 (Table 5). The Bonferroni post-hoc test showed that there was a significant difference when the absence of patent stump group was compared with the presence of reflux group (p = 0.017) and also comparing the partially patent stump group to the presence of reflux group (p = 0.032) (Table 6).

**Table 5:** Results of the ANOVA test of the relationship between LEED administered and closure of the GSV at the SFJ.

**Population**	**Sum of squares**	**df**	**Mean square**	**Z**	**p**
Intergroup	1453.727	2	726.863	3.978	0.021
Intragroup	27409.025	150	182.727		
Total	28862.751	152			

ANOVA, analysis of variance; df, degrees of freedom; SFJ, saphenofemoral junction; LEED, linear intravenous energy density; GSV, great saphenous vein.

**Table 6:** Mean LEED values and standard deviations for groups with absence of patent stump, partially patent stump, and presence of reflux at the SFJ.

**Anatomic reference**	**n**	**Mean (J/cm)**	**Standard deviation (J/cm)**
Absence of patent stump	41	72.59^b^	12.18
Partially patent stump	109	70.38^b^	12.71
Presence of reflux	3	49.90^a^	44.80
Total	153	70.57	13.77

SFJ, saphenofemoral junction; LEED, linear intravenous energy density; Different letters denote results with significant difference according to the Bonferroni test.

With relation to the type of fiber used and the LEED administered, there was a trend towards significant difference, according to the Levine test, with p = 0.084. According to this test, the linear fiber needed a higher LEED, of 73.30, compared to mean LEED of 69.21 for the radial fiber. When saphenous closure and presence of reflux were compared by type of fiber used, there was no significant difference (p = 0.174), according to the chi-square test.

## DISCUSSION

Chronic venous insufficiency can be defined as a condition of the superficial and/or deep venous system. The disease’s prevalence has been growing worldwide and increases with age. A study conducted with a population from Botucatu, in the Brazilian state of São Paulo, reported a 35.5% prevalence of varicose veins.^12^^-^ 14

Currently, studies investigating EVLA are attempting to identify safer ways of employing it, with fewer adverse effects. They analyze variables such as the wavelength and energy employed, the velocity at which the laser is applied, and different types of optical fibers.^10^

The laser light emitted from the generator causes a thermal reaction in which the laser energy delivered is captured by the intracellular water in the endothelial cells of the saphenous vein. This mechanism of action can be regulated by physical parameters, such as wavelength, method of energy administration, and quantity of energy per surface area.^15^^,^ 16

The level of energy absorbed is extremely important, since the vessel will only retract once it reaches a certain level and, if this is not achieved, the vein may remain open or recanalize.^17^^-^ 19 Conversely, an excessive amount of energy could injure adjacent tissues, such as nerves and lymph vessels. LEED is one of the most prominant variables during the procedure, and some studies have correlated energy density with favorable or unfavorable results.^13^^,^ 20 ^-^ 22

The term LEED was first used in 2005 and became the reference for calculation of the energy used in the procedure because of its simplicity and the possibility of standardization. The formula used takes into account the power used and the velocity at with the optical fiber is tractioned.^12^

In the lower limbs assessed in this study, mean LEED was 70.57 J/cm and it was observed that LEED exceeding this mean was necessary to achieve effective thermal ablation at the SFJ. The higher energy level provoked partial or complete obliteration at the SFJ and an absence of reflux. Similar results were observed by Proebstle et al.^17^

## CONCLUSIONS

At 30 days follow-up of patients treated for CVI under local anesthesia with EVLA, it was observed that at the SFJ using a LEED exceeding a mean of 70.57 J/cm was associated with better results, with higher rates of obliteration and absence of reflux, in relation to lower LEED. There was no significant difference in the thigh segment. This prompts the conclusion that for this segment it is not necessary to employ such a high energy level in order to achieve the desired results, and it is therefore possible to employ a lower LEED to reduce the chance of complications.

Although these results are compatible with the literature, further studies are needed with data specifically relating to the LEED applied at the SFJ and in the thigh, and their relationship with the incidence of paresthesia.
