# Supplementary material for: Influence of linear intravenous energy density on the success of intravenous laser ablation for treatment of chronic venous insufficiency
Source: J Vasc Bras. 2019 Aug 29;18:e20190009. doi: 10.1590/1677-5449.190009 (PMC6718192; doi:10.1590/1677-5449.190009)
Supplement: Translation

# Translation: Influência da densidade de energia endovenosa linear no sucesso da termoablação endovenosa a *laser* como tratamento da insuficiência venosa crônica

**Authors:** Luiza de Freitas Heineberg, Adriana Buechner de Freitas Brandão, Filipe Carlos Caron, Jaqueline Beirith, Walter Junior Boim de Araujo

**Affiliations:** 1 Faculdades Pequeno Príncipe, Curso Superior de Medicina, Curitiba, PR, Brasil.; 2 Instituto da Circulação - Excelência em Angiologia, Cirurgia Vascular e Endovascular, Departamento de Cirurgia, Curitiba, PR, Brasil.

**Keywords:** insuficiência venosa, laser, veia safena, varizes

## Abstract

**Contexto:**

Mostra-se relevante a elucidação da influência da densidade de energia endovenosa linear (*linear endovenous energy density,* LEED) média no sucesso do tratamento da insuficiência venosa crônica por termoablação endovenosa a *laser* visando diminuir a morbidade desse método.

**Objetivos:**

Avaliar a influência da LEED média na prevalência de fechamento das safenas após 30 dias do procedimento.

**Métodos:**

Foram analisados 153 membros inferiores de 118 pacientes de um hospital referência, submetidos a termoablação com *laser* endovenoso 1470 nm sob anestesia local para o tratamento de insuficiência venosa crônica. Foi calculada a LEED média dos pacientes tratados para determinar se foi necessária uma energia maior que a média para o sucesso do tratamento.

**Resultados:**

Dos 153 membros inferiores submetidos ao tratamento, houve diferença significativa (p = 0,021) no fechamento da junção safeno-femoral relacionada à LEED média utilizada acima do joelho. Por outro lado, no segmento de coxa não houve diferença significativa.

**Conclusões:**

A LEED acima da média de 70,57 J/cm apresentou um índice maior de fechamento na junção safeno-femoral. Porém, no segmento de coxa, a densidade não influenciou o resultado, demonstrando que uma energia mais alta do que 70,57 J/cm tende a não ser necessária para o tratamento desse segmento.

## INTRODUÇÃO

A insuficiência venosa crônica (IVC) é uma doença comum no consultório do cirurgião vascular e ocorre em 25 a 33% das mulheres e em 10 a 20% dos homens na população adulta ocidental^1^. Está diretamente relacionada ao absenteísmo laboral e a gastos do sistema público de saúde^2^. Diante dessa importância socioeconômica, o estudo de novas técnicas que possam viabilizar o tratamento efetivo com melhores resultados tem sido cada vez mais valorizado, principalmente em relação ao índice de recidivas, a complicações e ao retorno precoce às atividades diárias^3^.

O tratamento da IVC compreende: medidas clínicas e mudança de hábitos de vida; escleroterapia com espuma e cirurgia de varizes através da técnica de mini-incisões; safenectomia por fleboextração; e métodos endoluminais como radiofrequência e termoablação a *laser (endovenous laser ablation,* EVLA)^4^^-^ 7.

As técnicas minimamente invasivas são alternativas ao tratamento convencional cirúrgico de *stripping* da veia safena magna (VSM) e vêm ganhando espaço na medicina^5^^,^ 6. Entre elas destaca-se a EVLA, com resultados favoráveis no que diz respeito ao índice de fechamento das safenas, à satisfação do paciente e à menor morbidade pós-procedimento^3^^,^ 8 ^,^ 9.

Diante da crescente valorização da EVLA como um método alternativo ao *stripping* para o tratamento da IVC^10^, este estudo foi desenvolvido com a intenção de elucidar qual a influência da densidade de energia endovenosa linear (*linear endovenous energy density,* LEED) no desfecho do procedimento após 30 dias.

## MÉTODOS

Trata-se de um estudo retrospectivo que avaliou prontuários de 193 pacientes com IVC de um hospital referência, submetidos a EVLA e reavaliados após 30 dias. O projeto foi aprovado pelo Comitê de Ética da nossa instituição, sob o registro nº 80030817.0.00005580 na Plataforma Brasil.

Os critérios de inclusão foram pacientes com IVC submetidos a EVLA desde a junção safenofemoral (JSF) até o joelho no HAC de 2011 até fevereiro de 2018. Já os critérios de exclusão foram: pacientes que não retornaram em 30 dias; pacientes com doença arterial periférica, síndromes de hipercoagulação grave e história de trombose venosa profunda ou de insuficiência venosa profunda; e pacientes com prontuários incompletos.

Foram então analisados 153 membros inferiores de 118 pacientes submetidos ao tratamento com EVLA para IVC. O procedimento iniciava-se com o paciente em decúbito dorsal. Era realizado um botão anestésico no local da punção e procedia-se com punção ecoguiada da VSM com Abocath^®^16, preferencialmente em um ponto distal à última veia tributária com refluxo, seguida de instalação de introdutor valvulado 6F. A seguir, a fibra ótica (radial ou linear) era introduzida e conectada a um gerador de energia a *laser* endovenoso com comprimento de onda padrão de 1470 nm e registrado na Agência Nacional da Vigilância Sanitária (80058580018).

Uma vez certificada a posição endoluminal na VSM a 2,5 cm distal à JSF, fazia-se a indução anestésica local tumescente ao longo de todo o percurso da VSM a ser tratada. Utilizava-se seringa ou bomba de infusão mecânica, com uma solução formada por 250 mL de soro fisiológico a 0,9% para 20 mL de lidocaína a 2% e adrenalina 1:100.000 com 4 mL de bicarbonato de sódio a 8,4% (Figura 1). Uma vez finalizada a anestesia, conferia-se novamente a posição da fibra a 2,5 cm da junção e iniciava-se o disparo do *laser* (Figura 2). A fibra era tracionada manualmente, de forma contínua, em direção caudal até o limite distal da safena a ser tratada^11^.

**Figura 1:** Início da indução anestésica intumescente com controle ecográfico.

**Figura 2:** Confirmação do posicionamento da fibra através de transiluminação (A) e controle ecográfico (B).

Após o procedimento, realizava-se curativo com compressão extrínseca no trajeto da VSM com chumaço de algodão e meia elástica 7/8 de alta compressão (30-40 mmHg). A alta hospitalar se dava no mesmo dia, depois de aproximadamente 2 horas de observação após o término da cirurgia, com prescrição de analgésicos e anti-inflamatórios não esteroidais por 5 dias. A meia elástica era mantida por 48 horas e retirada pelo próprio paciente. A partir do terceiro dia, o paciente vestia as meias durante o dia e as retirava durante o banho e para dormir. Embora não houvesse restrição à deambulação, recomendava-se cerca de 40 minutos de repouso com pernas elevadas a cada hora.

Os pacientes foram reavaliados aos 7 e 30 dias após a cirurgia através de exame físico e ecografia vascular com Doppler (Figura 3). Entre os fatores analisados destacam-se: presença ou ausência de parestesia, flebite e trombose venosa profunda no membro inferior tratado, assim como taxa de obliteração da VSM e ausência de refluxo.

**Figura 3:** Controle ecográfico realizado no 7º dia pós-operatório demonstrando ausência de fluxo na veia safena.

A avaliação ecográfica da JSF quanto a obliteração e refluxo seguiu as recomendações das diretrizes de prática clínica para pacientes com varizes e doenças venosas da *Society for Vascular Surgery* e da *American Venous Forum*. Considerou-se resultado positivo a ausência de coto pérvio e de refluxo. Além disso, os pacientes foram divididos em ausência de coto pérvio, coto parcialmente pérvio e presença de refluxo (Tabela 1).

**Tabela 1:** Classificação dos resultados do eco-Doppler na junção safenofemoral após a termoablação.

Patência	J0	Ausência de coto pérvio
	J1, J2, J3, J4, etc.	Junção com coto pérvio de 1, 2, 3, 4 cm, etc.
Refluxo	R+	Refluxo
	R-	Sem refluxo

### Análise estatística

Os dados dos prontuários foram coletados e armazenados em uma planilha do Microsoft Excel. A análise de dados foi realizada com o auxílio do programa computacional SPSS, versão 22.0.

Os resultados foram expressos através de estatística descritiva. A análise inferencial foi realizada por meio do teste qui-quadrado de Pearson, teste exato de Fisher, teste *t* de Student para as variáveis paramétricas e teste de Mann-Whitney para as variáveis não paramétricas. Os valores de p menores que 0,05 foram considerados significativos.

## RESULTADOS

Dos 193 prontuários analisados, 118 foram selecionados para análise e 153 membros inferiores foram avaliados. A maioria da população do estudo era do sexo feminino (55,9%) e apresentava sobrepeso [índice de massa corporal (IMC) médio de 29,67], e a idade média foi de 55,35 anos. Foi utilizada fibra radial dois anéis em 66,8% e linear em 33,2% dos pacientes, todas com 600 µ de diâmetro e potência de 6 watts. Os dados descritivos são apresentados abaixo em forma de tabelas referentes à idade e ao IMC (Tabela 2), ao sexo (Tabela 3) e a complicações (Tabela 4).

**Tabela 2:** Dados descritivos referentes à idade e ao IMC.

Análise estatística	Idade (anos)	IMC (kg/m^2^)
n	118	118
Média	52,35	29,67
Desvio padrão	11,74	5,57

IMC, índice de massa corporal.

**Tabela 3:** Dados descritivos referentes ao sexo.

Sexo	Frequência	Porcentagem
Feminino	66	55,9
Masculino	52	44,1
Total	118	100,0

**Tabela 4:** Dados descritivos referentes à frequência de complicações após o procedimento nos 118 pacientes.

Complicação	Frequência	Porcentagem
TVP	1	0,8
Parestesia	31	26,3
Flebite	16	13,5

TVP, trombose venosa profunda.

Houve diferença significativa entre os grupos (ausência de coto pérvio, coto parcialmente pérvio e presença de refluxo) com relação à LEED média aplicada, segundo a análise de variância (ANOVA), com p = 0,021 (Tabela 5). O pós-teste de Bonferroni mostra que houve diferença significativa quando comparados os grupos que apresentaram ausência de coto pérvio e presença de refluxo (p = 0,017), assim como quando comparados os grupos que apresentaram coto parcialmente pérvio e presença de refluxo (p = 0,032) (Tabela 6).

**Tabela 5:** Resultados do teste ANOVA quanto à relação entre a LEED aplicada e o fechamento da VSM na JSF.

População	Soma dos quadrados	df	Quadrado médio	Z	p
Entre grupos	1453,727	2	726,863	3,978	0,021
Nos grupos	27409,025	150	182,727		
Total	28862,751	152			

ANOVA, análise de variância; df, graus de liberdade; JSF, junção safenofemoral; LEED, *linear endovenous energy density*; VSM, veia safena magna.

**Tabela 6:** Valores médios e desvio padrão da LEED para os grupos que apresentaram ausência de coto pérvio, coto parcialmente pérvio e presença de refluxo na JSF.

Referência anatômica	n	Média (J/cm)	Desvio padrão (J/cm)
Ausência de coto pérvio	41	72,59^b^	12,18
Coto parcialmente pérvio	109	70,38^b^	12,71
Presença de refluxo	3	49,90^a^	44,80
Total	153	70,57	13,77

JSF, junção safenofemoral; LEED, *linear endovenous energy density*; Letras diferentes indicam resultados com diferença significativa pelo teste de Bonferroni.

Em relação ao tipo de fibra utilizado e ao LEED aplicado, houve uma tendência de diferença significativa, segundo o teste de Levine, com p = 0,084. Conforme o teste, para a fibra linear foi necessária uma LEED média mais alta, de 73,30, enquanto na fibra radial a LEED média foi de 69,21. Quando comparados o fechamento das safenas e a presença de refluxo com o tipo de fibra utilizada, não houve diferença significativa (p = 0,174), de acordo com o teste qui-quadrado aplicado.

## DISCUSSÃO

A IVC pode ser definida como uma alteração do sistema venoso superficial e/ou profundo. A prevalência da doença tem crescido mundialmente e aumenta com a idade. Um estudo feito com uma população de Botucatu, no estado de São Paulo, apontou uma prevalência de varizes de 35,5%^12^^-^ 14.

Atualmente, existe um direcionamento dos estudos relacionados ao EVLA em busca de formas mais seguras de utilizá-lo com menos efeitos adversos. Para isso, levam-se em conta dados como o comprimento de onda e a energia utilizada, a velocidade com que se aplica o *laser* e os diferentes tipos de fibras ópticas^10^.

A liberação do *laser* a partir de um gerador levará a uma reação térmica em que a energia do *laser* é entregue e captada pela água intracelular das células endoteliais da safena. Esse mecanismo de ação pode ser regulado através de parâmetros físicos, tais como comprimento de onda, tipo de administração de energia e quantidade de energia por área de superfície^15^^,^ 16.

O nível de energia que será absorvido é de extrema importância, uma vez que a retração do vaso só ocorre quando se atingir um determinado patamar; caso contrário, a veia poderá manter-se aberta ou recanalizar^17^^-^ 19. Por outro lado, um excesso de energia poderá lesionar os tecidos adjacentes, como nervos e vasos linfáticos. A LEED é uma das variáveis mais marcantes durante o procedimento, e alguns estudos correlacionam a densidade de energia a resultados favoráveis ou desfavoráveis^13^^,^ 20 ^-^ 22.

O termo LEED começou a ser utilizado em 2005 e tornou-se referência no cálculo da energia utilizada no procedimento devido à sua facilidade e possível padronização. A fórmula que o compõe leva em conta a potência e a velocidade de tração com que se traciona a fibra óptica^12^.

Nos membros inferiores avaliados neste estudo, a LEED média foi de 70,57 J/cm, e observou-se que, para se obter uma termoablação efetiva na JSF, foi necessária uma LEED acima dessa média. A alta energia proporcionou a obliteração parcialmente completa ou completa na JSF e a ausência de refluxo, resultados também encontrados por Proebstle et al.^17^.

## CONCLUSÃO

Em 30 dias de seguimento dos pacientes submetidos a EVLA sob anestesia local para tratamento de IVC, mostrou-se que a utilização de uma LEED acima da média de 70,57 J/cm na JSF teve melhores resultados, com índices maiores de obliteração e ausência de refluxo, em relação a uma LEED menor. No segmento de coxa, não houve diferença significativa. Isso leva à conclusão de que, nesse segmento, não é necessária uma energia mais elevada para que se obtenha o resultado desejado, sendo possível, portanto, utilizar uma LEED mais baixa para diminuir a chance de complicações.

Apesar de os resultados serem compatíveis com a literatura, são necessários mais estudos com dados específicos sobre a LEED aplicada na JSF e no segmento de coxa, assim como a sua relação com a incidência de parestesia.
